# Dual trajectories of social participation and frailty in Chinese older adults: a longitudinal study based on CLHLS from 2008 to 2018

**DOI:** 10.3389/fpubh.2024.1401145

**Published:** 2024-09-04

**Authors:** Yiyun Bi, Jing Hu, Yilei Ma, Ping Yin, Peng Wang

**Affiliations:** ^1^Department of Epidemiology and Biostatistics, School of Public Health, Tongji Medical College, Huazhong University of Science and Technology, Wuhan, China; ^2^Operations Management Department, Union Hospital, Tongji Medical College, Huazhong University of Science and Technology, Wuhan, China; ^3^Division of Cardiothoracic and Vascular Surgery, Tongji Hospital, Tongji Medical College, Huazhong University of Science and Technology, Wuhan, China

**Keywords:** social participation, frailty, aged, longitudinal studies, group-based trajectory modeling

## Abstract

**Introduction:**

This study aimed to identify the dual trajectories of social participation (SP) and frailty index (FI) among Chinese older adults, and investigate common influential factors of both trajectories.

**Methods:**

Utilizing data from the Chinese Longitudinal Healthy Longevity Survey (CLHLS) 2008–2018 surveys, 1,645 individuals were analyzed. A group-based dual trajectory model and logistic regression were used to examine trajectories, their interrelations and shared influencing factors.

**Results:**

This study identified three SP, two FI trajectories and six distinct sub-groups of individuals. The study confirmed a long-term, interrelated relationship between two outcomes and identified some common factors. Compared to participants in the lower SP trajectory, those who followed the middle SP trajectory and higher SP trajectory had increased probabilities of belonging to the slow-growth FI trajectory (90.28 and 99.71%, respectively). And the participants in the slow-growth FI exhibited higher probabilities of belonging to the middle SP and the higher SP trajectory (37.64 and 25.34% higher, respectively) compared with those in the rapid-growth FI trajectory. Age, marital status, and drinking status were mutual factors associated with the dual trajectories.

**Discussion:**

The results showed significant associations between higher levels of frailty and lower levels of social participation. Related intervention policies should consider the dual trajectories and the common factors that underlie these trajectories of SP and FI.

## 1 Introduction

China has become an ageing society with the largest aging population globally (1). Achieving a healthy ageing society requires prioritizing the health needs of older persons and establishing optimal preparations for health care. Frailty, a critical indicator of treatment needs and its research have increased rapidly in Asia (2–4). It is strongly associated with the occurrence of depression (5), dementia (6), as well as overall and cause-specific mortality (7). Given the dynamic nature and varied developmental pathways of frailty (8), understanding its longitudinal change and influencing factors is essential for effective and timely intervention aimed at reducing risk. Several studies in Korea and Japan have explored the diverse trajectories of frailty among older adults, revealing the influence of factors like educational attainment, smoking, alcohol consumption, sleep, and marital status, while older adults who developed frailty and who remained frail were more likely to have cognitive impairment (9–12).

Social participation (SP), a central component of the World Health Organization’s approach to achieving healthy ageing (13), significantly impacts the mental and physical well-being of older adults (14) such as reducing the likelihood of dementia episodes (15) and depression (16). SP may follow distinct paths among different subgroups of older adults. Kawai et al. observed two trajectory patterns of the social interaction scores using large-scale longitudinal data from a cohort of older adults in Japan (17), and similar findings have been reported in China (18). Several studies in Asian countries have shown that age, sex, activities of daily living (ADL), having a chronic disease have an impact on social participation (17, 19, 20). Moreover, a study from Iran reported that educational level, income level and marital status influence the social participation of older people.

The majority of the current investigations have revealed a unidirectional relationship between social participation and frailty. An observational study conducted among older people in Japan revealed that ongoing engagement in social activities may lower the risk of frailty, specifically in oral function and depressive mood (21). Xie et al. (22) showed that promoting diverse forms of social interaction is a promising strategy to mitigating the burden associated with frailty in older individuals. Furthermore, embracing healthy habits and actively participating in social activities can help counteract the detrimental effects of declining health on overall life expectancy (23). While both cross-sectional and lagged social participation-frailty relationships have been observed (24), research exploring the potential direction of frailty level on social participation among Chinese older people remains limited.

Both frailty and social engagement status are instable that influence each other. Notably, the bidirectional, dynamic relationship between social participation and frailty remained under-explored in developing nations (25, 26). Hence, investigating the potential correlation between their paths and protective and risk variables can yield insights for healthcare and social services for older adults.

**Figure 1:**
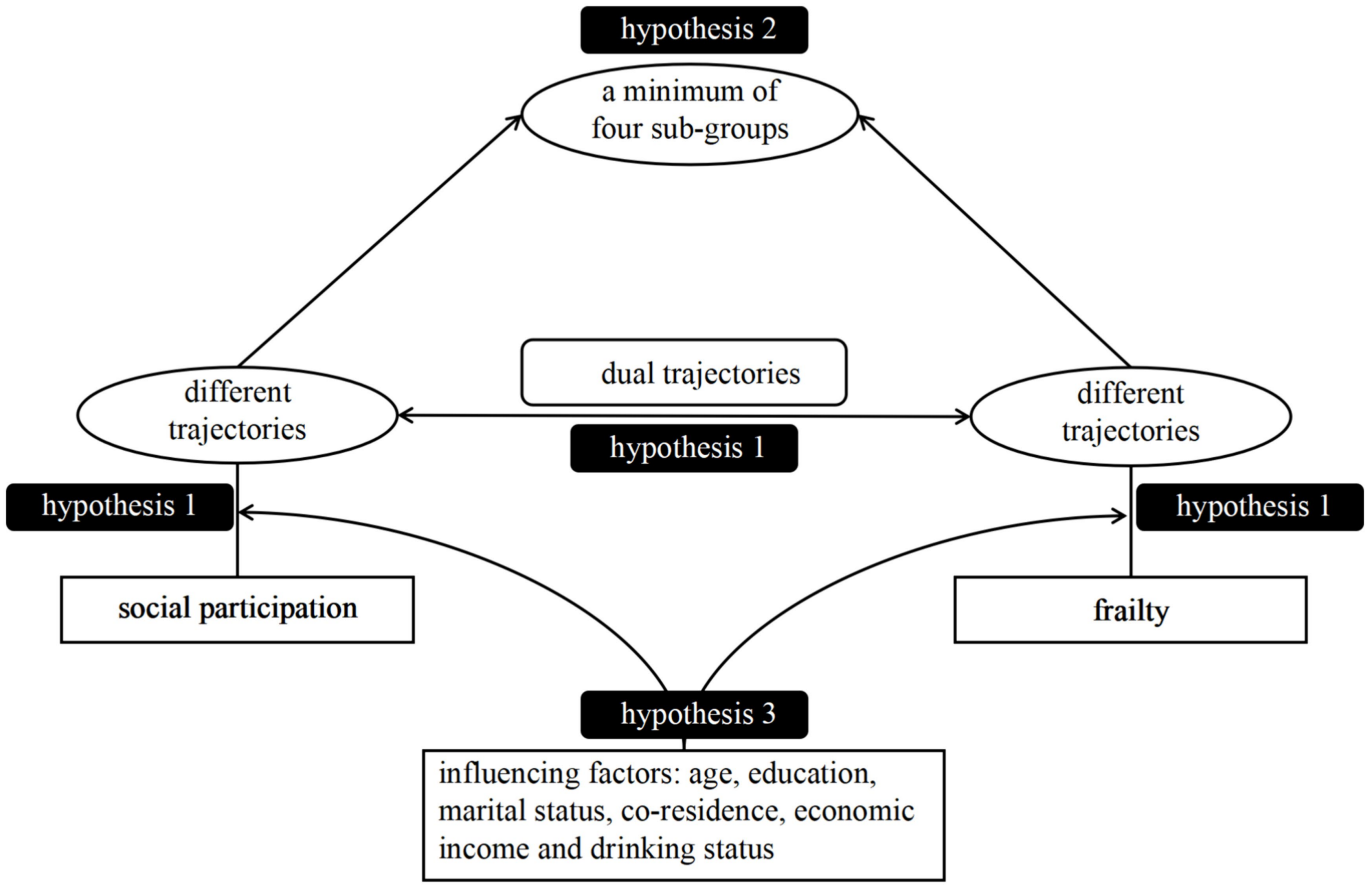
Conceptual framework of hypothetical model.

The current study investigated the trajectories of social participation and frailty index in a cohort of older Chinese adults from 2008 and 2018. We conducted a comprehensive analysis of the long-term and interconnected patterns between SP (social participation) and FI (frailty index) trajectories over a certain period. Additionally, we defined distinct sub-groups based on the combined trajectories of SP and FI. Ultimately, we examined the factors influencing the later dual trajectories of SP and FI. Figure 1 illustrates our conceptual framework and three hypotheses derived from the above review:

*H1:* We hypothesized that there will be distinct trajectories of both social participation and frailty index. Lower SP trajectories is associated with higher FI trajectories within each subgroup, and vice versa.

*H2:* We hypothesized the existence of a minimum of four sub-groups, determined by the dual trajectories.

*H3:* We hypothesized that age, education, marital status, co-residence, economic income and drinking status were shared risk factors that could impact the dual trajectories of SP and FI among older adults.

## 2 Materials and methods

### 2.1 Data and participants

The Chinese Longitudinal Healthy Longevity Survey (CLHLS) is a nationwide repeated cross-sectional survey applying a multistage, stratified cluster sampling between 1998 and 2018 which covers 631 randomly selected cities of 23 provinces in mainland China. The survey began in 1998, with subsequent surveys being conducted every 3–4 years. Ethical approval for the study was obtained from the ethics committees of Peking University. All participants or their proxy respondents provided informed consent.

Data from four waves of CLHLS from 2008 to 2018 were utilized in our study. Firstly, participants older than 65 years old at the 2008 baseline were included, following the definition of older adults (27). Of those, we excluded individuals with missing in 2011/2014/2018 wave, missing SP, missing frailty, missing covariates. Finally, we included 1,645 individuals for complete cases analysis. More details on participant inclusion and exclusion can be found in Supplementary Figure S1.

### 2.2 Measures

#### 2.2.1 Assessment of social participation

The assessment of social involvement required utilizing a thorough questionnaire consisting of 10 items encompassing cognitive, physical, and social activities. This questionnaire has been widely utilized and has demonstrated its validity (25, 28). House work, growing vegetables or doing other field work, garden work, reading newspapers or books, raise domestic animals or pets, playing cards or mah-jongg, watching tv or listening to radio, participating in some social activities, traveling and exercising are all part of these activities. The specifics about the selection and allocation of the indicators can be found in Supplementary Table S1. The social involvement indicator has a cumulative score of 34, with higher scores indicating greater levels of social participation.

#### 2.2.2 Assessment of frailty

The phenotypic approach and the frailty index (FI) are the predominant methodologies employed for evaluating an individual’s frailty status. The frailty level of participants was determined using the Frailty Index (FI) based on the Rockwood technique in this study (29). There exists 38 variables concerning chronic diseases, cognitive and physical function, activities of daily life, visual and hearing status, as well as the number of illnesses suffered in the past 2 years. Of those, we utilized the validated Chinese version of the Mini Mental State Examination (MMSE) to measure cognitive function (30). The score of each deficit was quantified into the 0 to 1 interval that was shown in Supplementary Table S2. Particularly, if the number of illnesses suffered in the past 2 years is more than 2, the variable has a score of 2. This method of calculation was established based on earlier studies conducted by CLHLS, and its validity and reliability has been demonstrated in these studies (31, 32). Reliability and validity tests of the FI and MMSE questionnaires were also conducted, using Cronbach’s alpha and KMO values as measures, respectively. The baseline data was employed for these calculations, revealing a Cronbach’s alpha value of 0.785 and a KMO value of 0.838 for FI. As for MMSE, both the Cronbach’s alpha value and the KMO value stood at 0.920. Consequently, the reliability and validity of the questionnaire are acceptable. In our study, each participant’s total FI score, ranging from 0 to 1, was determined by the ratio of the total sum of scores to the number of deficits included. A Frailty Index (FI) with less than 30 elements was classified as missing (33), while a FI more than 0.25 indicates frailty (34).

#### 2.2.3 Covariates

Building upon the existing extensive literature concerning the social engagement patterns and vulnerability of older Chinese individuals as previously documented (9–12, 17–20, 25, 32, 35), various covariates were assessed at baseline, encompassing sociodemographic characteristics [age, gender, residence, education, marital status, economic income], lifestyle behaviors [drinking, smoking, sleep duration], co-residence (alone, not alone). The age groups were categorized into 65–75 and ≥ 75 years and residence were defined as city and non-city. The levels of education were specified as uneducated (never being educated), primary school (being educated for 1–6 years), and middle school or above (being educated for 7 years or more). Current marital status included married, separation after marriage (separated, divorced, widowed) and unmarried. Low economic income was defined as the total household income of the previous year falling below the third quartile of all participants’. The smoking and drinking habits were categorized as never, previous, and current. We defined sleep as normal (5–10 h/day and no sleep disorder), excessive (>10 h/day) and insufficient (<5 h/day or having sleep disorder) (35).

#### 2.2.4 Analytic strategy

Similar to earlier research (18, 36), our analytical approach comprised three main components. Initially, group-based trajectory modeling (GBTM) was employed to ascertain trajectory patterns for two outcomes. Furthermore, both binary and multivariate logistic regression models were utilized to investigate the shared factors influencing their trajectories. Furthermore, a dual-trajectory model was employed to establish a connection between the complete longitudinal progression of social participation and frailty.

GBTM represents a specific implementation of finite mixture models that relies on non-parametric and semi-parametric statistical technique. This model assumes the existence of heterogeneity and explore the development trends of different groups over time. The optimal trajectory groups are expected to exhibit the following characteristics to the greatest extent possible (37): the smaller absolute Bayesian Information Criteria (BIC) value; the bigger change in BIC; the value of the Average Posterior Probability (APP) > 0.7; Odds of Correct Classification (OCC) > 5; Proportions per class ≥ 5%; Relative entropy ( $E_{k}$ ) is close to 1; parsimony; interpretability of groups. The “PROC TRAJ” command of SAS 9.4 was used.

The dual trajectory model offers a clear and simply comprehensive statistical description of the developmental connections between two desired outcomes. It can further be employed to uncover hidden groups of individuals who follow similar patterns across two indicators of interest (37). The dual model also offers the conditional and joint probabilities of belonging to each trajectory group, illustrating the combination or interaction between these two developmental variables (38). Additionally, the quantity of optional trajectory groups in the dual model commonly corresponds to those identified in the univariate models (38).

A two-tailed *p* value < 0.05 was used to determine statistical significance. The analyses were performed using SAS 9.4 version and SPSS 27 for Windows.

## 3 Results

### 3.1 Sample characteristics at baseline

Supplementary Table S3 shows the characteristics of the study sample. A total of 1,645 older adults had the mean (standard deviation, SD) of 74.73 (7.45) years at baseline, of which 48% were men and 52% were women. 13.71 (5.60) and 0.12 (0.06) were separately the mean (SD) social participation score and the mean (SD) FI score. Approximately 13% participants lived in the city but 87% did not. There were 59% of individuals with no education and 38% of them had primary education. Nearly 59% older adults among the participants were in current married status and 40% had separated after marriage. Most of them did not reside alone (85%), had low or middle economic income (75%), never drank (64%), never smoked (62%), and had normal sleep (80%).

### 3.2 Trajectories of social participation among older adults

**Figure 2:**
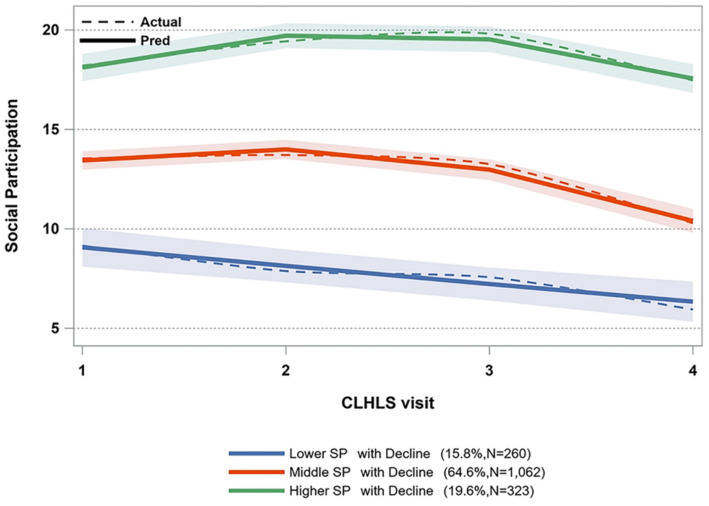
Trajectories of social participation.

We used four sequential models, varying from two-class to five-class models, with zero-order, linear, and quadratic specifications to determine the most appropriate model. The fitness information of different models is displayed in Supplementary Tables S4, S5. While the absolute BIC values of the four-class and five-class models were lower than that of the three-class model, the proportion of one group in the four-class and five-class models did not meet the 5% criterion. Consequently, considering the model’s interpretability and simplicity, we opted for the three-class model as the most suitable base model. Figure 2 illustrates the three paths of social engagement. As time progressed, it was observed that 15.8% of the older individuals exhibited a lower SP with a declining trajectory, whereas 64.5% displayed a middle SP with a declining trajectory, and 19.6% showed a higher SP with a declining trajectory.

### 3.3 Multivariate logistic regression of SP trajectories

The results of the multivariate logistic regression analysis, as presented in Table 1, indicate that individuals aged over 75 years demonstrated a reduced likelihood of 71.1 and 85.1% to be part of the middle SP group and higher SP group, respectively, when compared to the middle SP group. Individuals residing in either urban or rural areas exhibited considerably reduced likelihood of engaging in medium and higher levels of social participation. When comparing older people without education to those being educated for 1 ~ 6 years, the probabilities of belonging to the higher SP group were 3.17 times greater. And for those who received education of 7 years or more, the probability of belonging to the higher SP group was 6.809 times greater. Divorced older adults had a 49% lower probability of being in the medium SP trajectory and a 55.2% lower probability of being in the higher SP trajectory compared to the lower SP trajectory. Unmarried older adults were 77.7% less likely to be in the middle SP group than in the lower SP group. Participants who did not live alone were unlikely to be in the higher SP, in contrast with the lower SP. Middle or high income, drinking at present made older adults 107 and 85.7% more likely to be in the higher SP groups, respectively, than in the lower SP group.

**Table 1:** The logistic regression of SP trajectories.

Variables	Middle SP with Decline vs. Lower SP with decline			Higher SP with Decline vs. Lower SP with decline		
	OR		95% CI	OR		95% CI
		Lower	Upper		Lower	Upper
**Gender (ref. = Male)**						
Female	0.779	0.515	1.178	0.827	0.488	1.400
**Age, years (ref. = 65 ~ 75)**						
>75	0.289***	0.211	0.396	0.149***	0.098	0.228
**Residence (ref. = City)**						
Town or Rural	0.554*	0.312	0.982	0.164***	0.088	0.307
**Education level, years (ref. = 0)**						
1 ~ 6	1.226	0.872	1.724	3.170***	2.014	4.990
≥7	1.275	0.675	2.408	6.809***	3.360	13.800
**Marital status (ref. = In marriage)**						
Separation after marriage	0.510***	0.362	0.719	0.448***	0.280	0.714
Not in marriage	0.223*	0.068	0.735	0.230	0.044	1.199
**Co-residence (ref. = Alone)**						
Not alone	0.733	0.487	1.104	0.467*	0.258	0.845
**Economic income (ref. = Low)**						
Middle or high	1.292	0.894	1.868	2.070**	1.323	3.241
**Drinking status (ref. = Never)**						
Past	1.406	0.855	2.310	1.276	0.675	2.410
Present	1.226	0.801	1.878	1.857*	1.109	3.109
**Smoking status (ref. = Never)**						
Past	0.754	0.455	1.249	1.136	0.611	2.111
Present	1.130	0.707	1.806	1.393	0.792	2.451
**Sleep duration (ref. = Normal)**						
Excessive	0.704	0.339	1.464	1.205	0.466	3.115
Insufficient	1.670	1.670	1.670	1.670	1.670	1.670

OR, odds ratio; CI, confidence interval; *p < 0.05; **p < 0.01; ***p < 0.001.

### 3.4 Trajectories of frailty index among older adults

**Figure 3:**
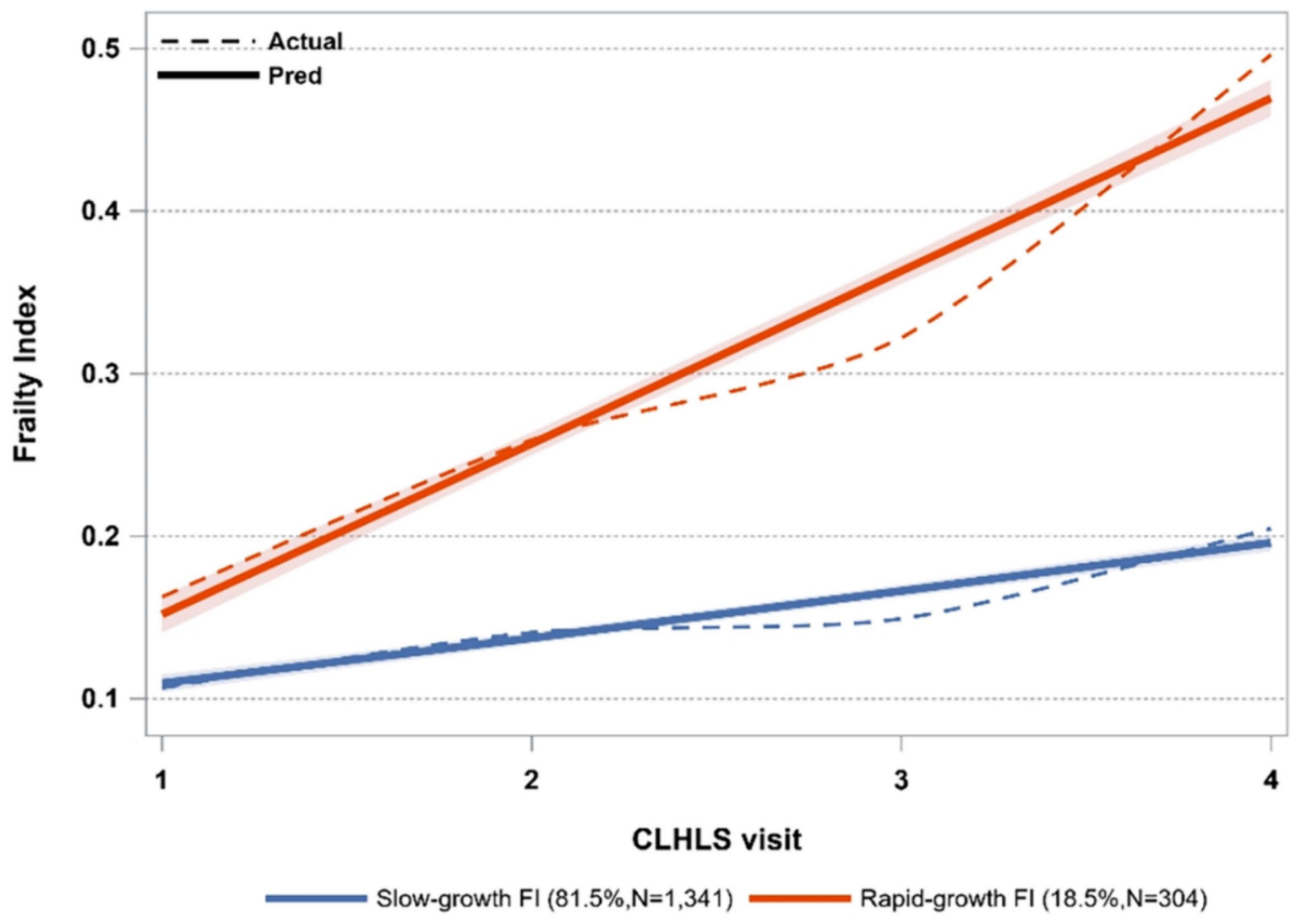
Trajectories of frailty index.

Model fitting was conducted through a systematic adjustment of the number of groups pertaining to the frailty index, ranging from two to five. The fitting parameters obtained from these analyses are presented in Supplementary Tables S6, S7. The two-class model exhibited the lowest absolute BIC value, satisfying additional criteria as well. Therefore, the two-class model was selected as the ultimate FI model. Figure 3 displays two paths of the frailty index. The initial category, characterized by a gradual increase in FI, comprised 81.5% of the total sample size (*N* = 1,341). The second class exhibited a swiftly expanding FI trend, encompassing 18.5% of the sample (*N* = 304).

### 3.5 Logistic regression of FI trajectories

The Logistic regression analysis of various FI trajectories is presented in Table 2. A higher likelihood of older women belonging to the rapid-growth FI group, compared to the slow-growth FI group, was observed to be 1.905 times. Similarly, participants aged over 75 had a 3.691 times greater likelihood of being in the rapid-growth FI category compared to those with slow-growing FI. Older people with only primary education and a current drinking habit had a likelihood of 34.7 and 35% lower, respectively, to be in the rapid growth FI trajectory compared to the slow-growth FI trajectory. Furthermore, post-marital separation and inadequate sleep were associated with a 46.1 and 48.4% higher likelihood, respectively, of older people being included in the rapid-growth FI groups compared to the slow-growth FI group.

**Table 2:** The logistic regression of FI trajectories.

Variables	Rapid-growth FI vs. Slow-growth FI		
	OR	95% CI	
		Lower	Upper
**Gender (ref. = Male)**			
Female	1.905**	1.282	2.829
**Age, years (ref. = 65 ~ 75)**			
>75	3.691***	2.757	4.941
**Residence (ref. = City)**			
Town or Rural	0.675	0.455	1.002
**Education level, years (ref. = 0)**			
1 ~ 6	0.653**	0.473	0.901
≥7	0.705	0.420	1.185
**Marital status (ref. = In marriage)**			
Separation after marriage	1.461*	1.060	2.013
Not in marriage	1.157	0.246	5.444
**Co-residence (ref. = Alone)**			
Not alone	1.264	0.854	1.870
**Economic income (ref. = Low)**			
Middle or high	0.754	0.540	1.052
**Drinking status (ref. = Never)**			
Past	0.996	0.639	1.551
Present	0.650*	0.428	0.987
**Smoking status (ref. = Never)**			
Past	1.550	0.976	2.462
Present	0.950	0.607	1.487
**Sleep duration (ref. = Normal)**			
Excessive	1.434	0.713	2.883
Insufficient	1.484*	1.060	2.078

OR, odds ratio; CI, confidence interval; *p < 0.05; **p < 0.01; ***p < 0.001.

### 3.6 Dual trajectories of social participation and frailty index

Panel A of Table 3 displays the conditional probability for each FI trajectory. The probabilities for the lower SP, middle SP, and higher SP groups collectively sum up to 100%. Older adults in the lower SP trajectory were most likely to belong to the rapid-growth FI trajectory (77.29%) and least likely to belong to the slow-growth trajectory (22.71%). Compared to participants in the lower SP trajectory, those who followed the middle SP trajectory and the higher SP trajectory had increased probabilities of belonging to the slow-growth FI trajectory (90.28 and 99.71%, respectively).

**Table 3:** Dual trajectory model for social participation and frailty index.

Social Participation	A: Probability of social participation conditional on frailty index	
	Frailty index	Conditional probability
Lower SP with decline	Slow-growth FI	22.71%
	Rapid-growth FI	77.29%
Middle SP with decline	Slow-growth FI	90.28%
	Rapid-growth FI	9.72%
Higher SP with decline	Slow-growth FI	99.71%
	Rapid-growth FI	0.29%

	B: Probability of frailty index conditional on social participation	
	Social participation	Conditional probability
Slow-growth FI	Lower SP with decline	4.65%
	Middle SP with decline	69.69%
	Higher SP with decline	25.66%
Rapid-growth FI	Lower SP with decline	67.63%
	Middle SP with decline	32.05%
	Higher SP with decline	0.32%

	C: Joint probability of social participation and frailty index	
Social participation	Slow-growth FI	Rapid-growth FI
Lower SP with decline	8.27%	7.54%
Middle SP with decline	53.98%	10.58%
Higher SP with decline	19.27%	0.36%

The conditional probabilities of belonging to each of the SP groups, given the frailty index groups, are displayed in Panel B. The probabilities for the slow-growth FI and rapid-growth FI groups each added up to 100%. When compared with older adults in the rapid-growth FI trajectory, those in the slow-growth FI exhibited higher probabilities of belonging to the middle SP and the higher SP trajectory (37.64 and 25.34% higher, respectively). These results suggest that rapid FI is associated with lower levels of SP within each SP subgroup.

Panel C illustrates the dual probabilities of participants in each of the SP and FI trajectories, where the six probabilities all added up to 100%. The group with slow-growth FI reporting middle SP had the highest probability (53.98%), followed by the group with slow-growth FI reporting higher SP (19.27%). The probability of the group with rapid-growth FI and higher SP was the lowest (0.36%).

## 4 Discussion

Our study investigated different trajectories of social participation and frailty state and the dynamic links between these trajectories of two outcomes. It was revealed that higher levels of frailty and lower levels of social participation exhibited significant bidirectional relationships, with age, education level, marital status, and drinking status potentially influencing the dual trajectories among Chinese older adults.

We have classified three unique trajectories of social participation (SP) among Chinese older people, each corresponding to different levels. A significant proportion of individuals (64.5%) aligns with the trajectory of medium SP with decline, spanning from 10 to 15, which is less than half of the total score. This suggests that a large portion of the older people in China exhibit a low level of social participation, potentially stemming from inadequate pension benefits. The Chinese government should encourage older adults to take part in social activities more frequently while providing more social resources. While the studies conducted in other Asian countries, such as Japan (17) and Korea (39), identified different numbers of SP trajectories, the trend observed in each trajectory closely mirrored our results, showing a gradual decline over time. At the same time, the two studies with larger sample sizes facilitated a more detailed grouping based on different levels of social participation, resulting in differences in the number of trajectories. Consistent with previous studies (26), this study identified two distinct FI trajectories.

Moreover, bidirectional longitudinal relationships between SP and FI were examined, a relationship that has not been thoroughly investigated in previous literature. Within each grouping, older people following a trajectory of decreasing lower SP were more likely to be included in the rapid-growth FI trajectory. Similarly, those involved in the rapid-growth FI trajectory had a higher likelihood of being in the lower SP with a decline. And these results confirm existing one-way evidence from studies in other East Asian countries. For example, Abe et al. reported that participating in social activities could improve the frailty status of older adults in Japan (40). A subsequent study involving older people found an association between functional disability and the number and types of social participation activities (41). Therefore, a strong correlation exists between decreased social participation (SP) and higher frailty status, consistent with prior research on the simultaneous and delayed connections between SP and frailty in older individuals (24). These results may be attributed to various factors. Social involvement offers social connections, increased possibilities for physical activity (42) and access to role-based support (43), which can enhance both physical and psychological well-being, thereby alleviating the negative effects of frailty. Simultaneously, the frailty index encompasses several aspects such as chronic illness, cognitive impairments, and functional restrictions, indicating that individuals with a high level of frailty are likely to have restricted participation in social activities (44). The above results partially validated our first supposition.

The present study identified six discrete subgroups of individuals displaying a combination of SP and FI trajectories, partially validating our initial hypothesis 2. Each of those groupings should be implemented with targeted intervention strategies in order to enhance their health status and overall quality of life. In the case of the rapid-growth of FI combined with higher SP trajectory group, there is a need to strongly emphasize interventions aimed at decreasing FI. A progressive exercise program combined with nutrition intervention led to enhancements in frailty status (45), indicating the necessity for increased vigilance from the community and family members toward the psychological well-being of older people to reduce adverse emotions (46). However, for the slow-growing FI with the lower or middle SP trajectory groups, enhancing the SP level is essential. Our suggestion is to focus on SP interventions, such as raise awareness regarding the importance of social engagement, providing multiple channels of social activities, conducting lectures on social etiquette, and enhancing expression skills for older adults (47). Individuals in the lower or middle SP who are experiencing rapid growing in frailty require more attention, which necessitates the integration of SP and interventions targeting frailty.

It is imperative to explore the influential factors, as clinical practice could focus on addressing risk factors and leveraging protective factors to create a supportive environment that caters to the diverse needs of older adults with varying trajectories of SP and FI. In our study, we explored the influencing factors of both SP and FI utilizing logistic regression, where the results partially confirmed hypothesis 3. Age emerges as a risk factor for lower SP and high FI, aligning with findings from previous studies (20, 48), underscoring the importance of focusing on the situation of older seniors (75+). Our research, in alignment with prior studies (48), indicated that older adults with primary education are more likely to belong to the slow-growth FI and higher SP trajectory. This could be attributed to the fact that low education could relate to worse choices regarding unhealthy behaviors, which leads to an increasing risk of frailty (49) and having less resources to take part in SP activities (18). A study conducted by Jung et al. found similar results indicating that older individuals in Korea with limited educational attainment exhibited a higher probability of belonging to the frailty trajectory characterized by escalating levels, potentially due to the impact of education on health behaviors (10). Furthermore, our study aligns with prior studies (50, 51) by demonstrating that individuals experiencing marital separation were more inclined to fall into the rapid-growth FI and lower SP trajectory. In a longitudinal study database developed by Nagai et al., it was evidenced that individuals who were widowed and faced economic stress were less likely to engage in regular activities, consequently displaying overall poorer health (51). China possesses a long history of alcohol culture, where drinking serves as a social lubricant associated with various social activities (52). Therefore, wine is the medium of socialization at the Chinese dinner table, which may be related to the level of social participation. Our findings indicated a heightened level of SP and lower frailty among older adults who currently consume alcohol, but the association between drinking and frailty exhibited inconsistencies compared to previous studies that documented various levels of frailty. One Mendelian randomization study indicated that there is no causal relationship between alcohol consumption and frailty (12). However, another analysis, including participants older than 55 years, revealed that people with heavier alcohol consumption had a reduced likelihood of frailty compared to non-drinkers (53). The volume of alcohol consumed or other confounding factors could potentially provide an explanation. Our study solely considered current or past drinking status, but the types of alcohol and drinking context may influence the results as well. Ortolá et al. reported that certain drinking patterns, especially drinking only with meals and the Mediterranean drinking pattern, are associated with a lower risk of frailty in older adults (54). Limited research exists in developing nations investigating the association between drinking patterns, frailty, and social engagement, aspects that will be addressed in our next plan.

This study had several strengths. Firstly, by utilizing nationally representative data on older adults in China, our findings provided valuable insights into the long-term patterns and associations between them. Secondly, this study explored common influencing factors associated with the dual trajectories of two variables which can give clinical practice to improve SP and frailty in older adults. However, it is important to acknowledge the study’s limitations. Firstly, selection bias may be present. Participants in the current study tended to be healthier than those excluded because our study spanned a decade. Secondly, our analysis focused on related baseline factors and did not encompass all indicators that may be associated with SP and FI trajectories. Thirdly, although we found the relation between the trajectories of the two outcomes, we should not interpret it as causality. Finally, the exploration of the dual trajectories of social participation and frailty in the older population is currently in its initial stage, and further researches should delve into the deeper mechanism of the two and the existence of unknown superimposed effects. Studies about the influence of lifestyle factors such as drinking patterns, dietary patterns on frailty and social participation are also of interest.

## 5 Conclusion

This study investigated significant connections between increased frailty levels and reduced social engagement, offering insights into healthcare and social services. To address the global ageing situation, intervention policies aiming at the dual trajectories and common underlying factors of SP and FI should be considered to enhance their effectiveness.

## Data Availability

Publicly available datasets were analyzed in this study. This data can be found at: https://opendata.pku.edu.cn/dataverse/CHADS.
