# Supplementary material for: Dual trajectories of social participation and frailty in Chinese older adults: a longitudinal study based on CLHLS from 2008 to 2018
Source: Front Public Health. 2024 Sep 4;12:1401145. doi: 10.3389/fpubh.2024.1401145 (PMC11408341; doi:10.3389/fpubh.2024.1401145)
Supplement: Supplementary file 1 [file Table_1.DOCX]

Dual Trajectories of Social Participation and Frailty in Chinese Older Adults: A Longitudinal Study Based on CLHLS from 2008 to 2018

# Supplementary Figures and Tables

## Supplementary Figures

**CLHLS Survey in 2008**

Total 16564 participants(aged$\geq$65)

Excluded: N=14915

- Missing in 2011/2014/2018: N=14197
- Missing SP: N=83
- Missing frailty: N=635

**Complete cases analysis**

**(N=1645)**

Missing covariates: N=4

1649 participants

met the inclusion criteria

**Supplementary Figure 1.** Flowchart of sample selection and preprocessing (n represents sample size).

## Supplementary Tables

Supplementary Table 1. Selection and assignment of the social participation indicators

| Variables | Values |
| --- | --- |
| do you read newspapers/books at present? | almost everyday=4; not daily, but once for a week=3; not weekly, but at least once for a month=2; not monthly, but sometimes=1; never=0 |
| do you play cards/mah-jongg at present? | almost everyday=4; not daily, but once for a week=3; not weekly, but at least once for a month=2; not monthly, but sometimes=1; never=0 |
| do you watch tv or listen to radio at present ? | almost everyday=4; not daily, but once for a week=3; not weekly, but at least once for a month=2; not monthly, but sometimes=1; never=0 |
| do you do house work at present? | almost everyday=4; not daily, but once for a week=3; not weekly, but at least once for a month=2; not monthly, but sometimes=1; never=0 |
| do you do garden work? | almost everyday=4; not daily, but once for a week=3; not weekly, but at least once for a month=2; not monthly, but sometimes=1; never=0 |
| do you grow vegetables & do other field work at present? | almost everyday=4; not daily, but once for a week=3; not weekly, but at least once for a month=2; not monthly, but sometimes=1; never=0 |
| do you raise domestic animals/pets at present? | almost everyday=4; not daily, but once for a week=3; not weekly, but at least once for a month=2; not monthly, but sometimes=1; never=0 |
| do you take part in some social activities at present? | almost everyday=4; not daily, but once for a week=3; not weekly, but at least once for a month=2; not monthly, but sometimes=1; never=0 |
| times of traveling beyond home county/city in the past two years | never=0; more than once=1 |
| exercise or not at present? | yes=1; no=0 |

Supplementary Table 2. Selection and assignment of frailty index.

| Dimension | Variables | Denoted as the condition of the defect | Score |
| --- | --- | --- | --- |
| Cognitive function | The Chinese version of the Mini-Mental State Examination (MMSE) contains 24 questions | ≤23 | 1 |
|  |  | >23 | 0 |
| Chronic disease | Hypertension, diabetes, tuberculosis, heart disease, stroke/cerebrovascular disease, bronchitis/asthma, cancer, arthritis, pressure ulcers, stomach/duodenal ulcers, Parkinson's disease | Suffer from the disease | Each disease=1 |
|  |  | else | 0 |
| Activities of daily life | feeding | without assistance | 0 |
|  |  | with some help | 0.5 |
|  |  | need feeding | 1 |
|  | bathing | without assistance | 0 |
|  |  | one part assistance | 0.5 |
|  |  | more than one part assistance | 1 |
|  | dressing | without assistance | 0 |
|  |  | need assistance for trying shoes | 0.5 |
|  |  | assistance in getting clothes and getting dressed | 1 |
|  | toileting | without assistance | 0 |
|  |  | assistance in cleaning or arranging clothes | 0.5 |
|  |  | don't use toilet | 1 |
|  | indoor transferring | without assistance | 0 |
|  |  | with assistance | 0.5 |
|  |  | bedridden | 1 |
|  | continence | without assistance | 0 |
|  |  | occasional accidents | 0.5 |
|  |  | incontinent | 1 |
|  | able to go outside to visit neighbors? | yes | 0 |
|  |  | a little difficult | 0.5 |
|  |  | unable to do so | 1 |
|  | able to go shopping by yourself? | yes | 0 |
|  |  | a little difficult | 0.5 |
|  |  | unable to do so | 1 |
|  | able to make food by yourself? | yes | 0 |
|  |  | a little difficult | 0.5 |
|  |  | unable to do so | 1 |
|  | able to wash clothes by yourself? | yes | 0 |
|  |  | a little difficult | 0.5 |
|  |  | unable to do so | 1 |
|  | able to walk one kilometer? | yes | 0 |
|  |  | a little difficult | 0.5 |
|  |  | unable to do so | 1 |
|  | able to carry 5kg weight? | yes | 0 |
|  |  | a little difficult | 0.5 |
|  |  | unable to do so | 1 |
|  | able to crouch and stand three times? | yes | 0 |
|  |  | a little difficult | 0.5 |
|  |  | unable to do so | 1 |
|  | able to take public transportation? | yes | 0 |
|  |  | a little difficult | 0.5 |
|  |  | unable to do so | 1 |
| Physical function | hand behind neck | both hands | 0 |
|  |  | right or left hand | 0.5 |
|  |  | neither hand | 1 |
|  | hand behind lower back | both hands | 0 |
|  |  | right or left hand | 0.5 |
|  |  | neither hand | 1 |
|  | hold-up arms | right of left arm | 0 |
|  |  | two arms | 0.5 |
|  |  | neither arm | 1 |
|  | able to stand up from sitting in a chair? | yes, without using hands | 0 |
|  |  | yes, using hands | 0.5 |
|  |  | no | 1 |
|  | able to pick up a book from the floor? | yes, standing | 0 |
|  |  | yes, sitting | 0.5 |
|  |  | no | 1 |
| Self-rated health | self-reported health | very good | 0 |
|  |  | good | 0.25 |
|  |  | so so | 0.5 |
|  |  | bad | 0.75 |
|  |  | very bad | 1 |
|  | do you feel any change of your health since last year | much better | 0 |
|  |  | a little better | 0.25 |
|  |  | no change | 0.5 |
|  |  | a little worse | 0.75 |
|  |  | much worse | 1 |
| Visual status | visual function: can you see the break in the circle? | can see and distinguish | 0 |
|  |  | can see only | 0.5 |
|  |  | can't see or blind | 1 |
| Hearing status | was interviewee able to hear? | yes, without hearing aid | 0 |
|  |  | yes, but needs hearing aid | 0.5 |
|  |  | partly, despite hearing aid or no | 1 |
| Psychological function | feel fearful or anxious | always | 1 |
|  |  | often | 0.75 |
|  |  | sometimes | 0.5 |
|  |  | seldom | 0.25 |
|  |  | never | 0 |
|  | the health of interviewee rated by interviewer | surprisingly or relatively healthy | 0 |
|  |  | moderately ill | 0.5 |
|  |  | very ill | 1 |
|  | times of suffering from serious illness within the past two years | none | 0 |
|  |  | one | 1 |
|  |  | more than two | 2 |

Supplementary Table 3. Descriptive characteristics at baseline

| Variables | Total (n = 1645) |
| --- | --- |
| Age, Mean (SD) | 74.73(7.45) |
| Social participation, Mean (SD) | 13.71(5.60) |
| Frailty Index, Mean (SD) | 0.12(0.06) |
| Gender |  |
| Male | 788(0.48) |
| Female | 857(0.52) |
| Residence |  |
| City | 218(0.13) |
| Town or Rural | 1,427(0.87) |
| Education level, years |  |
| 0 | 771(0.47) |
| 1~6 | 631(0.38) |
| ≥7 | 243(0.15) |
| Marital status |  |
| In marriage | 966(0.59) |
| Separation after marriage | 662(0.40) |
| Not in marriage | 17(0.01) |
| Co-residence |  |
| Alone | 242(0.15) |
| Not alone | 1,403(0.85) |
| Economic income |  |
| Low | 410(0.25) |
| Middle or High | 1235(0.75) |
| Drinking status |  |
| Never | 1,062(0.64) |
| Past | 209(0.13) |
| Present | 374(0.23) |
| Smoking status |  |
| Never | 1,026(0.62) |
| Past | 232(0.14) |
| Present | 387(0.24) |
| Sleep duration |  |
| Normal | 1,308(0.80) |
| Excessive | 54(0.03) |
| Insufficient | 283(0.17) |

Continuous variables are shown as mean (SD) and categorical variables are shown as frequency (%).

Low economic income：total income of household lower than the first quartile of all participants’ last year

Table S4. Evaluation metrics for fitting effectiveness of different SP trajectory models.

| Group | Avepp(%) | OCC | P_j_(%) | π_j_(%) | BIC^#2^ | _#△_BIC^#2^ | E_k_ |
| --- | --- | --- | --- | --- | --- | --- | --- |
| 2Group (2 2 ) | 91.07-87.08 | 5.8-11.8 | 64.92-35.08 | 63.66-36.34 | -20538.76 |  | 0.652 |
| 3Group (1 2 2 ) | 80.28-84.72-84.64 | 18.2-3.6-20.9 | 15.81-64.56-19.64 | 18.32-60.82-20.86 | -20454.48 | 84.29 | 0.657 |
| 4Group (1 2 2 0 ) | 78.45-82.28-77.55-79.22 | 21.9-3.6-9.8-103.7 | 12.16-59.88-25.35-2.61 | 14.24-56.11-26.10-3.55 | -20447.15 | 7.33 | 0.666 |
| 5Group (1 0 2 2 0 ) | 76.08-55.74-64.08-73.13-78.21 | 16.3-4.7-3.2-8.8-106.6 | 16.17-14.16-41.09-26.08-2.49 | 16.36-21.10-35.64-23.64-3.26 | -20448.74 | -1.60 | 0.527 |
| AIC(Akaike information criterion), BIC#2(Bayesian Information Criterion), *E_k_*(Relative entropy), Avepp (Average posterior probability). | | | | | | | |

Table S5. Evaluation of the fitting effect of each trajectory group within the SP trajectory models.

| Group | Class | Parameter | *β* | *SE* | *t* | *P* |
| --- | --- | --- | --- | --- | --- | --- |
| *2Group* | Class.1 | Intercept | 10.48680 | 0.46548 | 22.529 | 0.00000 |
|  |  | Linear | 1.76826 | 0.42567 | 4.154 | 0.00003 |
|  |  | Quadratic | -0.56913 | 0.08393 | -6.781 | 0.00000 |
|  | Class.2 | Intercept | 13.85749 | 0.64868 | 21.363 | 0.00000 |
|  |  | Linear | 3.95095 | 0.59071 | 6.688 | 0.00000 |
|  |  | Quadratic | -0.87309 | 0.11660 | -7.488 | 0.00000 |
| *3Group* | Class.1 | Intercept | 9.98666 | 0.51937 | 19.228 | 0.00000 |
|  |  | Linear | -0.96587 | 0.15669 | -6.164 | 0.00000 |
|  | Class.2 | Intercept | 11.30745 | 0.48380 | 23.372 | 0.00000 |
|  |  | Linear | 2.92047 | 0.44704 | 6.533 | 0.00000 |
|  |  | Quadratic | -0.78817 | 0.08748 | -9.010 | 0.00000 |
|  | Class.3 | Intercept | 14.72953 | 0.86857 | 16.958 | 0.00000 |
|  |  | Linear | 4.28123 | 0.78999 | 5.419 | 0.00000 |
|  |  | Quadratic | -0.89321 | 0.15517 | -5.756 | 0.00000 |
| *4Group* | Class.1 | Intercept | 9.31076 | 0.59204 | 15.727 | 0.00000 |
|  |  | Linear | -0.88975 | 0.17966 | -4.952 | 0.00000 |
|  | Class.2 | Intercept | 11.15700 | 0.52468 | 21.264 | 0.00000 |
|  |  | Linear | 2.59506 | 0.48723 | 5.326 | 0.00000 |
|  |  | Quadratic | -0.74586 | 0.09586 | -7.781 | 0.00000 |
|  | Class.3 | Intercept | 12.97086 | 0.89545 | 14.485 | 0.00000 |
|  |  | Linear | 4.45543 | 0.78057 | 5.708 | 0.00000 |
|  |  | Quadratic | -0.93745 | 0.15326 | -6.117 | 0.00000 |
|  | Class.4 | Intercept | 22.25921 | 0.62578 | 35.570 | 0.00000 |
| *5Group* | Class.1 | Intercept | 9.59854 | 0.54125 | 17.734 | 0.00000 |
|  |  | Linear | -0.91809 | 0.17034 | -5.390 | 0.00000 |
|  | Class.2 | Intercept | 12.67465 | 0.64711 | 19.587 | 0.00000 |
|  | Class.3 | Intercept | 10.50357 | 0.77691 | 13.520 | 0.00000 |
|  |  | Linear | 4.17425 | 0.84487 | 4.941 | 0.00000 |
|  |  | Quadratic | -1.17983 | 0.18745 | -6.294 | 0.00000 |
|  | Class.4 | Intercept | 12.85787 | 0.93848 | 13.701 | 0.00000 |
|  |  | Linear | 4.93957 | 0.89711 | 5.506 | 0.00000 |
|  |  | Quadratic | -1.04304 | 0.18132 | -5.752 | 0.00000 |
|  | Class.5 | Intercept | 22.41147 | 0.63092 | 35.522 | 0.00000 |
| Significant level was set at P≤0.05. | | | | | | |

Table S6. Evaluation metrics for fitting effectiveness of different FI trajectory models.

| Group | *Avepp(%)* | *OCC* | *P_j_(%)* | *π_j_(%)* | *BIC^#2^* | *_#△_BIC^#2^* | *E_k_* |
| --- | --- | --- | --- | --- | --- | --- | --- |
| *2Group (1 1 )* | 98.20-92.38 | 12.4-53.3 | 81.52-18.48 | 81.46-18.54 | 6113.11 |  | 0.901 |
| *3Group (1 2 1 )* | 97.22-87.76-92.41 | 12.2-30.3-166.5 | 74.35-18.91-6.75 | 74.08-19.11-6.81 | 6425.20 | 312.09 | 0.891 |
| *4Group (1 1 2 1 )* | 96.76-82.16-87.48-94.92 | 12.1-33.0-53.2-350.5 | 71.98-11.49-11.55-4.98 | 71.09-12.23-11.61-5.06 | 6577.75 | 152.56 | 0.886 |
| *5Group (1 1 2 1 1 )* | 85.89-79.29-84.27-95.88-95.75 | 402.0-22.6-46.1-10.7-413.2 | 1.52-13.01-10.94-69.48-5.05 | 1.49-14.46-10.42-68.46-5.17 | 6625.15 | 47.39 | 0.879 |
| AIC(Akaike information criterion), BIC#2(Bayesian Information Criterion), *E_k_*(Relative entropy), Avepp (Average posterior probability). | | | | | | | |

Table S7. Evaluation of the fitting effect of each trajectory group within the FI trajectory models.

| Group | Class | Parameter | *β* | *SE* | *t* | *P* |
| --- | --- | --- | --- | --- | --- | --- |
| *2Group* | Class.1 | Intercept | 0.07480 | 0.00294 | 25.442 | 0.00000 |
|  |  | Linear | 0.03021 | 0.00113 | 26.735 | 0.00000 |
|  | Class.2 | Intercept | 0.04424 | 0.00646 | 6.848 | 0.00000 |
|  |  | Linear | 0.10630 | 0.00250 | 42.520 | 0.00000 |
| *3Group* | Class.1 | Intercept | 0.08262 | 0.00281 | 29.402 | 0.00000 |
|  |  | Linear | 0.02481 | 0.00108 | 22.972 | 0.00000 |
|  | Class.2 | Intercept | 0.21845 | 0.01549 | 14.103 | 0.00000 |
|  |  | Linear | -0.11901 | 0.01701 | -6.996 | 0.00000 |
|  |  | Quadratic | 0.04311 | 0.00371 | 11.620 | 0.00000 |
|  | Class.3 | Intercept | 0.10827 | 0.01207 | 8.970 | 0.00000 |
|  |  | Linear | 0.10997 | 0.00530 | 20.749 | 0.00000 |
| *4Group* | Class.1 | Intercept | 0.07778 | 0.00272 | 28.596 | 0.00000 |
|  |  | Linear | 0.02503 | 0.00102 | 24.539 | 0.00000 |
|  | Class.2 | Intercept | 0.12945 | 0.01027 | 12.605 | 0.00000 |
|  |  | Linear | 0.05299 | 0.00336 | 15.771 | 0.00000 |
|  | Class.3 | Intercept | 0.29825 | 0.01723 | 17.310 | 0.00000 |
|  |  | Linear | -0.23330 | 0.01702 | -13.707 | 0.00000 |
|  |  | Quadratic | 0.06992 | 0.00355 | 19.696 | 0.00000 |
|  | Class.4 | Intercept | 0.07823 | 0.01261 | 6.204 | 0.00000 |
|  |  | Linear | 0.12922 | 0.00480 | 26.921 | 0.00000 |
| *5Group* | Class.1 | Intercept | 0.37067 | 0.03035 | 12.213 | 0.00000 |
|  |  | Linear | -0.01241 | 0.01144 | -1.085 | 0.27805 |
|  | Class.2 | Intercept | 0.08419 | 0.00833 | 10.107 | 0.00000 |
|  |  | Linear | 0.06162 | 0.00327 | 18.844 | 0.00000 |
|  | Class.3 | Intercept | 0.31338 | 0.01734 | 18.073 | 0.00000 |
|  |  | Linear | -0.25005 | 0.01687 | -14.822 | 0.00000 |
|  |  | Quadratic | 0.07389 | 0.00347 | 21.294 | 0.00000 |
|  | Class.4 | Intercept | 0.07825 | 0.00272 | 28.768 | 0.00000 |
|  |  | Linear | 0.02397 | 0.00106 | 22.613 | 0.00000 |
|  | Class.5 | Intercept | 0.07158 | 0.01102 | 6.495 | 0.00000 |
|  |  | Linear | 0.13057 | 0.00410 | 31.846 | 0.00000 |
| Significant level was set at P≤0.05. | | | | | | |
